# *Notice to Readers:* Forthcoming Correction and Republication of the Report “Deaths and Years of Potential Life Lost From Excessive Alcohol Use — United States, 2011–2015”

**DOI:** 10.15585/mmwr.mm6935a7

**Published:** 2020-09-04

Recently, the authors of the report “Deaths and Years of Potential Life Lost From Excessive Alcohol Use — United States, 2011–2015” (*1*) informed *MMWR* Editors that some results were inaccurate as a result of a data input error that occurred during an update to the online Alcohol-Related Disease Impact application (*2*), which was used in the study. This error resulted in an overall underestimate of average annual alcohol-attributable deaths by 1,862 and years of potential life lost by 79,844 for the United States during 2011–2015. On September 3, 2020, corrections were made in the online Alcohol-Related Disease Impact application to the alcohol-attributable fractions for five acute causes of death: drownings, fall injuries, fire injuries, firearm injuries, and homicide. The updated national and state estimates are now available in the Alcohol-Related Disease Impact application (*2*). The authors conducted a reanalysis and verification of the data, and a revised report will be published in the coming weeks.
